# A Review: Design from Beta Titanium Alloys to Medium-Entropy Alloys for Biomedical Applications

**DOI:** 10.3390/ma16217046

**Published:** 2023-11-05

**Authors:** Ka-Kin Wong, Hsueh-Chuan Hsu, Shih-Ching Wu, Wen-Fu Ho

**Affiliations:** 1 Department of Chemical and Materials Engineering, National University of Kaohsiung, Kaohsiung 81148, Taiwan; m1075616@mail.nuk.edu.tw; 2 Department of Dental Technology and Materials Science, Central Taiwan University of Science and Technology, Taichung 40601, Taiwan; hchsu@ctust.edu.tw (H.-C.H.); scwu@ctust.edu.tw (S.-C.W.)

**Keywords:** biomedical alloy, high-entropy alloys, medium-entropy alloy, metastable, β-Ti alloys

## Abstract

β-Ti alloys have long been investigated and applied in the biomedical field due to their exceptional mechanical properties, ductility, and corrosion resistance. Metastable β-Ti alloys have garnered interest in the realm of biomaterials owing to their notably low elastic modulus. Nevertheless, the inherent correlation between a low elastic modulus and relatively reduced strength persists, even in the case of metastable β-Ti alloys. Enhancing the strength of alloys contributes to improving their fatigue resistance, thereby preventing an implant material from failure in clinical usage. Recently, a series of biomedical high-entropy and medium-entropy alloys, composed of biocompatible elements such as Ti, Zr, Nb, Ta, and Mo, have been developed. Leveraging the contributions of the four core effects of high-entropy alloys, both biomedical high-entropy and medium-entropy alloys exhibit excellent mechanical strength, corrosion resistance, and biocompatibility, albeit accompanied by an elevated elastic modulus. To satisfy the demands of biomedical implants, researchers have sought to synthesize the strengths of high-entropy alloys and metastable β-Ti alloys, culminating in the development of metastable high-entropy/medium-entropy alloys that manifest both high strength and a low elastic modulus. Consequently, the design principles for new-generation biomedical medium-entropy alloys and conventional metastable β-Ti alloys can be converged. This review focuses on the design from β-Ti alloys to the novel metastable medium-entropy alloys for biomedical applications.

## 1. Introduction

In the field of biomedical materials, the quest for ideal implant materials that simultaneously possess excellent mechanical properties, corrosion resistance, and biocompatibility remains a paramount challenge. Conventional 316L stainless steel, Co-based alloys, and Ti-based alloys have long served as the industry standards for hip and knee joint replacements [1]. However, the ever-increasing demand for these materials, driven by an aging population and expanding medical applications, has exposed their limitations.

316L stainless steel, while widely used, exhibits insufficient corrosion resistance and wear resistance, leading to issues like stress corrosion and the release of allergenic Ni and toxic Cr ions [2]. Similarly, the Co–Cr–Mo alloy exhibits excellent corrosion resistance attributed to the formation of a passive Cr_2_O_3_ passivation layer on its surface. However, when this passivation layer wears off, its corrosion resistance significantly decreases [3]. The corrosion products of Co–Cr–Mo alloy are also more biologically toxic than those of 316L stainless steel [4]. Ti–6Al–4V ELI alloy exhibits excellent mechanical strength, lower elastic modulus, and good biocompatibility. However, the Al ions in the alloy are suspected to be associated with symptoms such as peripheral neuropathy, osteomalacia, and Alzheimer’s disease [5]. Additionally, V ions can induce cytotoxicity [5]. Moreover, when the elastic modulus of implant materials significantly exceeds that of human cortical bone, it can lead to stress shielding, causing bone tissue atrophy and implant failure [6]. The elastic moduli of 316L stainless steel (210 GPa), Co–Cr–Mo (240 GPa), and Ti–6Al–4V ELI (110 GPa) are much higher than that of the human cortical bone (about 30 GPa) [6], making them less suitable as a biomedical implant.

In recent years, metastable β-Ti alloys have gained attention for their low elastic moduli and outstanding properties, which encompass high specific strength, low elastic modulus, excellent fatigue resistance, high toughness, exceptional corrosion resistance, and remarkable biocompatibility [7]. These alloys present a promising alternative to traditional implant materials, addressing the issue of stress shielding effectively. Their exceptional blend of characteristics renders them as excellent candidates for orthopedic and dental implant applications. However, metastable β-Ti alloys with low elastic moduli often exhibit concomitantly reduced mechanical strength [7]. Furthermore, the advent of High-Entropy Alloys (HEAs) has revolutionized alloy design by incorporating multiple primary elements, offering new possibilities for orthopedic and dental implant applications [8]. However, challenges, such as compositional segregation and a high elastic modulus, still persist.

This review explores the emerging developments in the field of biomedical materials, with a focus on metastable β-Ti alloys and HEAs. We discuss the ongoing efforts to address the limitations of traditional implant materials and the promising advancements in achieving high-strength, low-modulus materials for biomedical applications. These novel materials aim to provide improved performance and extend the lifespan of biomedical implants, ultimately benefiting patients and the medical community.

## 2. Metastable β-Phase Biomedical Ti Alloys

### 2.1. Phase Stability Calculations

Ti alloy systems are primarily categorized into α (Hexagonal), α + β (hexagonal + body-centered cubic), and β (body-centered cubic) types, with the phase structure depending on the types of solute elements present in the alloy. Alloys with approximately 1–2 wt% of β stabilizers and around 5–10 wt% of the β phase are classified as near-α alloys. Alloys containing higher levels of β stabilizers, resulting in 10–30 wt% of the β phase in the microstructure, are categorized as α + β alloys. Alloys with even higher β stabilizers, allowing the β phase retention through rapid cooling, are termed as metastable β-Ti alloys [9]. Commonly used β-stabilizing elements include Nb, Ta, Mo, and W, etc. Additionally, Zr, Sn, and Si are neutral elements. It is important to note that Zr and Sn are identified as β-stabilizing elements in the Ti–Nb alloy system [10,11].

The commonly used theories for the phase stability of Ti alloys are: (1) bond order (Bo) and d-orbital energy level (Md), (2) valence electron concentration (VEC), (3) Mo equivalent ([Mo]_eq_), and (4) martensite start temperature (M_s_). The Bo-Md theory is based on DV-Xα-Cluster molecular orbital calculations, utilizing Bo and Md to predict the stability and phase transformation of Ti alloys. The calculation formulas for the Bo and Md values of Ti alloys are as follows [7]: Bo = ΣX_i_(Bo)_i_, Md = ΣX_i_(Md)_i_, where X_i_ is the atomic fraction of each element in the alloy, and (Bo)_i_ and (Md)_i_ are the Bo and Md values of alloy element i in β-type Ti alloy, respectively.

Ikehata et al. [12] used first-principles calculations to study the effect of VEC in binary Ti alloy systems on elastic modulus, suggesting that the VEC value of the alloy predicts its crystal structure. When the VEC value of the alloy is approximately 4.2–4.24, the alloy can reach the BCC metastable state and obtain a lower elastic modulus.

[Mo]_eq_ is an empirical formula derived from experimental results [13] that quantifies the contribution of each alloying element to the β-phase stability of Ti alloys. When the [Mo]_eq_ value is less than 10 wt% and close to 10 wt%, the alloy achieves a metastable β-phase, resulting in a lower elastic modulus [14]. The formula for calculating [Mo]_eq_ is as follows [13]: [Mo]_eq_ = 1.0 [Mo] + 0.74 [V] + 0.50 [W] + 0.39 [Nb] + 0.28 [Ta] + 2.2 [Fe] + 1.69 [Cr] + 0.85 [Cu] + 1.22 [Ni] + 1.57 [Co] + 1.69 [Mn] + 0 [Sn] + 0 [Zr] − 1.0 [Al] (wt%).

Furthermore, within the design process, adjusting the compositions of Ti alloys to shift the M_s_ closer to room temperature, a Ti alloy with a metastable β phase at room temperature can be obtained. The M_s_ temperature formula for binary Ti alloy systems is [15]: M_s_ (°C) = 1156 − 150[Fe] − 96[Cr] − 49[Mo] − 37[V] − 17[Nb] − 7[Zr] + 15[Al] (wt%).

### 2.2. Advantages of Metastable β-Ti Alloys

**Figure 1:**
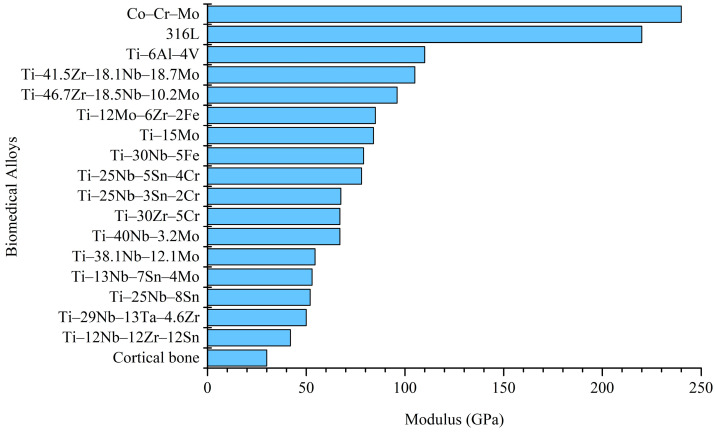
Comparison of the modulus among published biomedical metastable Ti alloys, conventional biomedical alloys, and cortical bone [17,18,19,20,21,22,23,24,25,26,27].

Among the developed biomedical Ti alloys, metastable β-Ti alloys typically exhibit lower elastic moduli [16]. Figure 1 presents a comparison of the elastic moduli of conventional biomedical metals (Co–Cr–Mo, 316L, and Ti–6Al–4V), metastable β-Ti alloys, and the human cortical bone [17,18,19,20,21,22,23,24,25,26,27]. Metastable β-Ti alloys with a low elastic modulus include Ti–12Mo–6Zr–2Fe [20] and Ti–15Mo [28], which have been included in ASTM standards.

In 1998, Niinomi [16] and Kuroda et al. [26] developed the Ti–29Nb–13Ta–4.6Zr (TNTZ) metastable β-Ti alloy with an elastic modulus of 65 GPa using the d-electron design method, with Bo and Md values of 2.878 and 2.462, respectively. Additionally, Ho et al.’s [14] study on Ti–Mo alloys found that when [Mo]_eq_ is below 10 wt% and close to 10 wt%, the alloy achieves a metastable β phase. Subsequently, in 2005, Hao et al. [29] developed the Ti–24Nb–4Zr–7.9Sn (TNZS) metastable β-Ti alloy with an elastic modulus of 42 GPa using the VEC theory, with a VEC value of 4.15. Hao et al. [30] reported that when the VEC of β-Ti alloys is within the range of 4.1 to 4.25, it may induce the formation of metastable α″ and ω phases. Suppression of these metastable phases (α″ and ω) can lead to obtaining a metastable β phase with an exceptionally low modulus.

In addition to mechanical performance, biomedical metals need to demonstrate excellent corrosion resistance and biocompatibility. Common biomedical β-Ti alloys are composed of toxic-free elements such as Ti, Zr, Nb, Ta, and Mo. A substantial body of literature has confirmed that the excellent corrosion resistance of β-type biomedical Ti alloys is attributed to the high stability of their oxidation passivation layer [31]. Taking Ti–Zr–Nb alloy as an example, the surface oxide layer is composed of oxides such as TiO_2_, ZrO_2_, Nb_2_O_3_, and Ta_2_O_3_ [31]. These oxides contribute significantly to the formation of a continuous and stable oxidation passivation layer on the alloy’s surface [31]. On the other hand, the biocompatibility of elements such as Ti, Zr, Nb, Ta, and Mo has been confirmed through in vitro cell culture tests [32,33]. Moreover, the Ti and Zr elements further promote the growth of fibrillar adhesions of osteoblasts [32].

The design of Ti alloys in the metastable β-phase achieves an ultra-low elastic modulus while maintaining good biocompatibility. However, in metastable β-Ti alloys, there is a trade-off between a low elastic modulus and high yield strength, resulting in low yield strength while maintaining a low elastic modulus [34]. Increasing the strength of the implant material not only contributes to applications in various hard tissues in the human body but also enhances the material’s fatigue resistance, reducing the likelihood of clinical failure and extending the life of the implant material [35]. Furthermore, increasing hardness helps overcome the poor wear resistance of conventional Ti alloys [36]. Therefore, the inadequate mechanical properties of conventional biomedical alloys have created an opportunity for the development of high-entropy alloys in the field of biomedical metal implants.

## 3. HEAs

### 3.1. Four Core Effects

**Figure 2:**
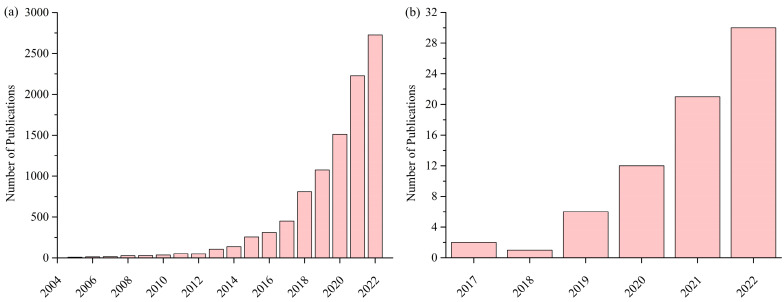
Number of international journal publications on (**a**) high-entropy alloys and medium-entropy alloys from 2004 to 2022 and (**b**) biomedical high-entropy alloys/medium-entropy alloys from 2017 to 2022 (Source: www.webofscience.com).

**Figure 3:**
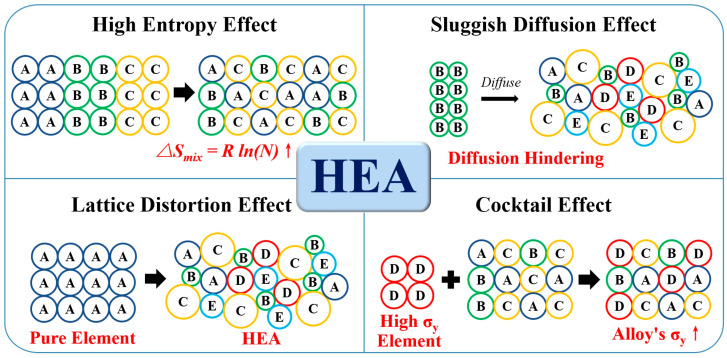
The four core effects of high-entropy alloys.

In 2004, Yeh et al. [37,38,39] revolutionized alloy design by proposing the concept of HEAs, wherein designs are no longer confined to a single primary element but instead incorporate five or more main elements. Since 2004, the number of international journal papers related to HEAs has continuously increased, reaching over 2500 publications in 2022 (Figure 2a). Recently, researchers have discovered the potential of utilizing HEAs in the biomedical field. The number of related international journal publications has significantly increased over the years, accumulating to over 70 articles by 2022 (Figure 2b). The four core effects of HEAs, briefly illustrated in Figure 3, contribute to the alloys’ outstanding properties.

#### 3.1.1. High Entropy Effect

The high entropy effect facilitates the mixing of various elements into a solid solution. In n-component HEA systems with equiatomic ratios, the formula for calculating the change in mixing entropy (ΔS_mix_) is as follows [37], according to Equation (1):

(1) $$\Delta S_{mix}=R\ln n$$

where R is the gas constant (R = 8.314 J/K·mol).

For equiatomic-ratio alloys composed of two, three, four, and five elements, the ΔS_mix_ values are 0.693R, 1.099R, 1.386R, and 1.609R, respectively. According to the formula for calculating Gibbs free energy (ΔG_mix_ = ΔH_mix_ − TΔS_mix_), a higher ΔS_mix_ in the alloy reduces the ΔG_mix_, making it easier for the alloy to form a single solid solution rather than intermetallic compounds during solidification.

#### 3.1.2. Sluggish Diffusion Effect

The sluggish diffusion effect of HEAs can be explained from a kinetic perspective. Substitutional atoms primarily diffuse through a vacancy mechanism. Each vacancy faces competition from atoms of different surrounding elements for diffusion. If an atom jumps into a vacancy site with low energy, subsequent jumps out of the site become more difficult. Conversely, if a vacancy site has high energy, the atom does not undergo diffusion. This phenomenon restrains the diffusion of alloying elements and leads to a slower diffusion process. This effect can prevent structural changes of the alloy at high temperatures, enhancing the thermal stability of the alloy [40].

#### 3.1.3. Lattice Distortion Effect

The lattice of HEAs is composed of multiple elements, and the different atomic sizes of elements leads to a highly distorted lattice, significantly affecting the properties of the alloy. Distortions in the lattice structure can enhance the solid solution strengthening effect; however, they can also impede the mobility paths of electrons and phonons, thereby reducing the electrical and thermal conductivity efficiency of the alloy [41].

#### 3.1.4. Cocktail Effect

The properties of HEAs are closely related to their constituent elements. Adding lighter elements can decrease the alloy density, and incorporating elements with stronger corrosion resistance enhances the alloy’s corrosion resistance. Furthermore, the additional effects resulting from interactions among elements are crucial in influencing the properties of the alloy. The combination of these effects is referred to as the “cocktail effect” [42].

### 3.2. Thermodynamic Parameters

Guo et al. [43] pointed out that the formation of a solid solution in HEAs requires satisfying thermodynamic parameters such as atomic size misfit (δ) and mixing enthalpy (ΔH_mix_), according to Equations (2) and (3), respectively. In addition, VEC can predict the phase structure of the solid solution, according to Equation (4). The formulas for these thermodynamic parameters are as follows [43]:

(2) $$\delta =\sqrt{\sum_{i=1}^{n}{c_{i}(1-r_{i}/\bar{r})}^{2}}$$

where c_i_ and r_i_ are the atomic percentage and atomic radius of the ith element, respectively.

(3) $$\Delta H_{mix}=4\sum_{i=1,i\neq j}^{n}\Delta H_{mix}^{AB}c_{i}c_{j}$$

where $\Delta H_{mix}^{AB}$ is the mixing enthalpy of the binary AB alloy.

(4) $$VEC=\sum_{i=1}^{n}c_{i}{\left(VEC\right)}_{i}$$

where (VEC)_i_ is the valence electron concentration of the ith element.

Guo et al. [43], through the integration of some studies on HEAs, inferred that the formation of a solid solution in the alloy is mainly influenced by three parameters: δ, ΔH_mix_, and ΔS_mix_. The formation of a solid solution requires satisfying the conditions 0 ≤ δ ≤ 8.5, −22 ≤ ΔH_mix_ ≤ 7 kJ/mol, and 11 ≤ ΔS_mix_ ≤ 19.5 J/(K·mol).

VEC can predict whether the formed solid solution of an alloy is in the FCC or BCC phase [43]. When VEC ≥ 8, the alloy is in the FCC phase, 6.87 ≤ VEC < 8 results in the FCC + BCC phase, and VEC < 6.87 leads to the BCC phase. Iijima et al. [44] suggested that [Mo]_eq_ can predict the phase structures of HEAs. The alloy exhibits the BCC phase when [Mo]_eq_ ≥ 11.8, 9.8 < [Mo]_eq_ < 11.8 results in a mixture of BCC + HCP phases, and [Mo]_eq_ ≤ 9.8 contributes to the HCP phase. They also stated that there is no significant correlation between [Mo]_eq_ and other thermodynamic parameters.

In recent years, researchers have developed some non-equiatomic medium-entropy alloys (MEAs) [45,46,47] and ternary MEAs [48,49,50]. Although the ΔS_mix_ of these new MEAs does not meet the conditions mentioned earlier (11 ≤ ΔS_mix_ ≤ 19.5 J/K·mol), the ΔH_mix_ of the alloys is close to or less than zero, allowing for the free energy (ΔG_mix_ = ΔH_mix_ − TΔS_mix_) of the alloys to be at a lower value, facilitating the formation of a solid solution.

## 4. Evolution of Biomedical High-Entropy Alloys (Bio-HEAs)

### 4.1. Equiatomic Bio-HEAs

Wang and Xu [51] first published a biomedical equiatomic Ti–Zr–Nb–Ta–Mo HEA in 2017. In selecting alloy elements, they referred to the equiatomic refractory alloys, Ta–Nb–Hf–Zr–Ti, Hf–Nb–Ti–Zr, Hf–Mo–Ta–Ti–Zr, and Hf–Mo–Nb–Ta–Ti–Zr with a single BCC phase, developed by Senkov et al. [52]. Wang and Xu utilized Ti–Zr–Nb–Ta–Mo as the base and excluded the Hf element due to its lower resistance to tribocorrosion in simulated body fluid [53]. They introduced the Mo element with high hardness and elastic modulus to the alloy, aiming to enhance its wear resistance [54]. The results showed that the as-cast Ti–Zr–Nb–Ta–Mo alloy exhibited better compressive yield strength (1330 MPa) than conventional biomedical alloys (Ti–6Al–4V, 316L S.S., and Co–Cr–Mo). Furthermore, Ti–Zr–Nb–Ta–Mo had comparable corrosion resistance to Ti–6Al–4V in the potentiodynamic polarization test. However, due to the significant differences in the melting points of the alloy elements, Ti–Zr–Nb–Ta–Mo experienced severe dendritic segregation, forming a dendritic and interdendritic structure. This substantial compositional segregation resulted in the main BCC_1_ and minor BCC_2_ phases with different lattice constants. Severe compositional segregation can have various adverse effects on the alloy, such as increased elastic modulus, reduced ductility, or decreased corrosion resistance.

Nagase et al. [55] investigated the effects of annealing on the microstructure, compositional segregation, mechanical properties, and biocompatibility of equiatomic Ti–Zr–Nb–Ta–Mo HEA. Post annealing, the alloy’s compression deformability increased from 4.5% to 7.5%. In cell culture tests, the survival rate of osteoblasts in annealed Ti–Zr–Nb–Ta–Mo was significantly higher than that of commercially pure Ti. However, prolonged high-temperature annealing (1000 °C for 168 h) only moderately improved the segregation issue in the alloy, unable to completely eliminate the minor BCC_2_ phase. Consequently, equiatomic HEAs face a severe compositional segregation issue, and extended annealing can provide only limited improvement. This segregation problem significantly impacts the alloy’s mechanical properties and corrosion resistance, making it unsuitable for biomedical applications.

### 4.2. Advantages of Non-Equiatomic Ti-Rich HEAs

#### 4.2.1. Improvement of Compositional Segregation

Nagase et al. [56] employed the FactSage simulation system and discovered that the formation of dual BCC phases (BCC_1_ + BCC_2_) in equiatomic Ti–Zr–Nb–Ta–Mo HEA is primarily due to the segregation of components during solidification. By adjusting the alloy composition and increasing the Ti content to lower the alloy’s melting point, they developed a non-equiatomic Ti_2.6_–Zr–Nb–Ta–Mo HEA that improved the segregation issue, successfully eliminating the minor BCC_2_ phase. Unfortunately, compositional segregation was still observed in the EPMA-WDS mapping results for Ti_2.6_–Zr–Nb–Ta–Mo.

Subsequently, Hori et al. [32] obtained similar results in the development of Ti_1.4_–Zr_1.4_–Nb_0.6_–Ta_0.6_–Mo_0.6_ using the FactSage simulation system. Although Ti_1.4_–Zr_1.4_–Nb_0.6_–Ta_0.6_–Mo_0.6_ exhibited a single BCC structure, severe compositional segregation akin to Ti_2.6_–Zr–Nb–Ta–Mo was observed in the EPMA-WDS mapping results. This indicates that utilizing simulation systems to avoid compositional segregation holds significant potential, and there is still considerable room for improvement.

In order to further develop an alloy with a low melting point, Nagase et al. [57] developed biomedical Ti–Zr–Hf–Cr_0.2_–Mo and Ti–Zr–Hf–Co_0.07_–Cr_0.07_–Mo HEAs based on a combination of the Ti–Nb–Ta–Zr–Mo and Co–Cr–Mo alloys. By adjusting the elemental proportions and incorporating lower-melting-point elements, they aimed to lower the melting point to below 2000 °C, making HEA production more cost-effective. Experimental results aligned with the design expectations. By introducing Cr and Co elements into the alloy, the alloy’s melting point was reduced, and the hardness of the alloy was enhanced through the formation of intermetallic compounds (Laves phase). The formation of the Laves phase is associated with the smaller atomic radii of the Co and Cr elements compared to other alloy elements and their lower ΔH_mix_ compared with other elements [58]. However, the Cr and Co elements might induce severe segregation in the alloy, and the presence of the Laves phase could potentially lead to adverse effects such as increased elastic modulus, reduced ductility, and decreased corrosion resistance in the alloy.

In 2021, Iijima et al. [44] developed biomedical HEAs, Ti_32.07_–Zr_27.58_–Hf_18.49_–Nb_12.70_–Ta_0.19_–Mo_8.97_, Ti_32.61_–Zr_28.58_–Hf_20.39_–Nb_12.05_–Ta_0.80_–Mo_5.57_, and Ti_28.33_–Zr_28.33_–Hf_28.33_–Nb_6.74_–Ta_6.74_–Mo_1.55_, by adjusting the thermodynamic parameters of the alloys. The results showed that reducing the VEC value of the alloy can improve the segregation of components, which is mainly due to the reducing contents of Nb, Ta, and Mo with high melting points and high VEC values. Additionally, the study indicated that [Mo]_eq_ could predict the phase structure of HEAs.

#### 4.2.2. Enhancing Mechanical Strength

Han et al. [59] developed a series of Ti_x_–Nb–Mo–Ta–W (x = 0, 0.25, 0.5, 0.75, and 1 in at%) HEAs and investigated the influence of different Ti contents on the mechanical properties of the alloys. The study found a significant increase in yield strength and compressive plasticity with increasing Ti content in the alloy. Specifically, Ti_1_–Nb–Mo–Ta–W showed a 31% increase in yield strength compared to Ti_0.25_–Nb–Mo–Ta–W. The literature suggests that the substantial increase in yield strength of the alloy is mainly attributed to the addition of Ti with a larger radius, which increases the lattice constant of Ti_x_–Nb–Mo–Ta–W, further enhancing the solid solution strengthening. Additionally, similar results were observed in the W–Nb–Mo–Ta–Zr_x_ (x = 0.1, 0.3, 0.5, and 1 in at%) HEAs system, where the addition of more Zr led to a significant increase in strength, hardness, and compressive plasticity due to solid solution strengthening, grain refinement, and dendritic structure strengthening [60].

#### 4.2.3. Improving Ductility

Hori et al. [32] designed two sets of non-equiatomic Ti–Nb–Ta–Zr–Mo HEAs: Ti_2-x_–Zr_2-x_–Nb_x_–Ta_x_–Mo_x_ and Ti_2-y_–Zr–Nb–Ta–Mo_y_. The results of compression tests indicated that the compressive deformability (>30%) of Ti_2-x_–Zr_2-x_–Nb_x_–Ta_x_–Mo_x_ (x = 0.6) and Ti_2-y_–Zr–Nb–Ta–Mo_y_ (y = 0.3, 0.5) was significantly higher compared to the equiatomic Ti–Nb–Ta–Zr–Mo alloy (~5%), demonstrating that a higher Ti content can improve the compressive deformability of the alloy.

Furthermore, Han et al. [59] pointed out that the grain boundary cohesion of Ti_x_–Nb–Mo–Ta–W can be improved with an increase in Ti content. The enhanced grain boundary cohesion effectively suppresses intergranular fracture, thereby enhancing the ductility of Ti_x_–Nb–Mo–Ta–W.

#### 4.2.4. Decrease in Modulus

Yang et al. [61] employed a statistical clustering formula approach to develop Ti_25_–Zr_25_–Hf_25_–Nb_12.5_–Ta_12.5_ and Ti_27.78_–Zr_27.78_–Hf_27.78_–Nb_8.33_–Ta_8.33_ HEAs, both of which exhibited low compressive moduli (56–68 GPa). Unfortunately, the report only briefly attributed the low compressive modulus of the alloys to their compositions rich in the Zr and Hf elements, lacking further detailed discussion.

#### 4.2.5. Enhancing Corrosion Resistance

In Section 2 on “Metastable β-phase Biomedical Ti Alloys”, it is explained that the excellent corrosion resistance of Ti alloys depends on the stability of the passive oxide layer on the surface, which is influenced by the material’s composition. Hence, in the design of HEAs with non-equiatomic ratios, more elements that enhance the stability of the passive oxide layer can be added, such as Zr, Nb, Ta, and Mo. For example, adding Nb to the alloy can inhibit the occurrence of activation and depassivation of the passive oxide layer [62]. Furthermore, incorporating more Zr elements can refine the grain size and reduce the degree of compositional segregation, thereby enhancing the stability of the passive oxide layer [63].

Hua et al. [64] reported on the corrosion behavior of Ti_x_–Zr–Nb–Ta–Mo (x = 0.5, 1, 1.5, and 2 in at%) in phosphate-buffered saline solution. The results showed that reducing the Ti content could lower the passivation current density. X-ray photoelectron spectroscopy results revealed that Ti_0.5_–Zr–Nb–Ta–Mo had the highest oxygen content in the passive oxide layer, indicating the formation of a thicker passive oxide layer on its surface. Consequently, the oxide passivation layer of Ti_0.5_–Zr–Nb–Ta–Mo demonstrated the highest stability.

#### 4.2.6. Enhancing Biocompatibility

**Figure 4:**
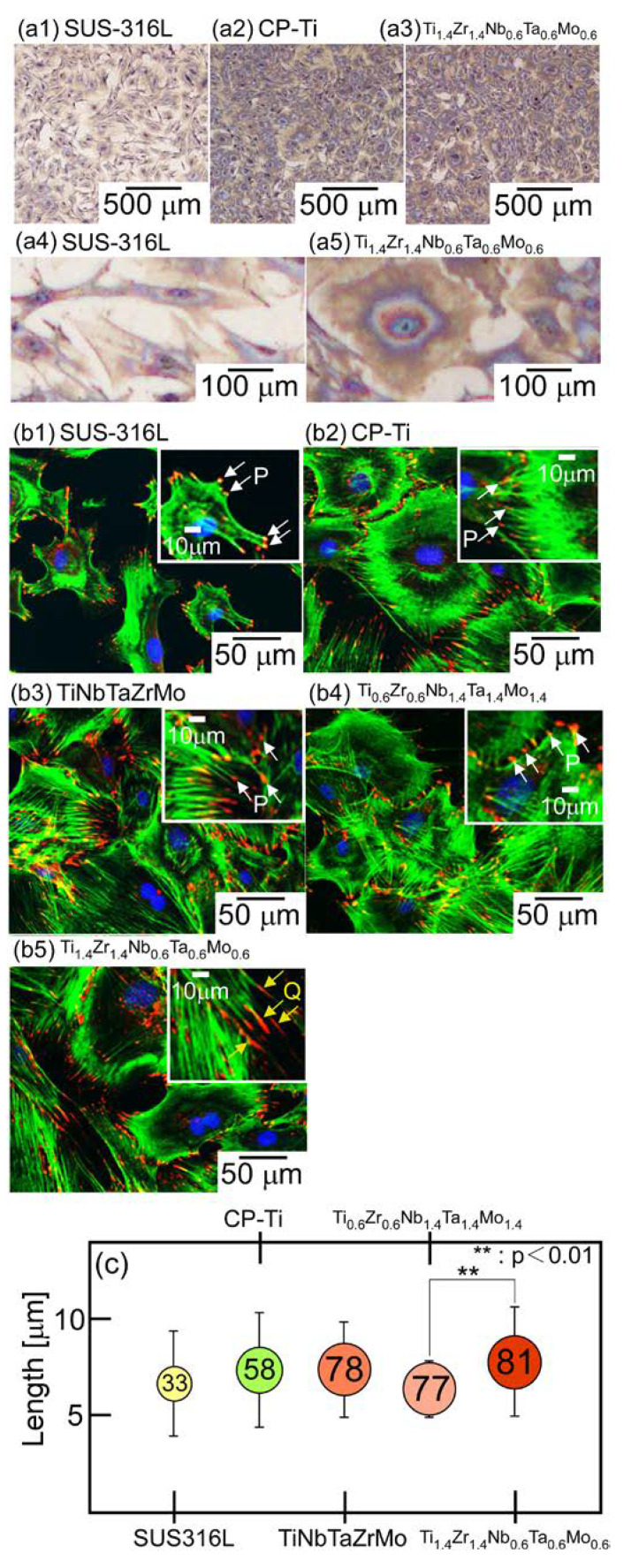
Biocompatibility of SUS–316L, CP-Ti, equiatomic Ti–Nb–Ta–Zr–Mo, and non-equiatomic Ti_(2−x)_–Zr_(2−x)_–Nb_x_–Ta_x_–Mo_x_ (x = 0.6, 1.4) (x = 0.6, 1.4) Bio-HEAs. (**a**) Giemsa staining images of osteoblasts, (**b**) fluorescent images of osteoblast adhesion, and (**c**) quantitative analysis of fibrillar adhesion size regulation [32]. (Reprinted with permission under the terms of the Creative Commons CC-BY license from Elsevier: Scr. Mater.)

Hori et al. conducted a study on the biocompatibility of Ti_(2−x)_–Zr_(2−x)_–Nb_x_–Ta_x_–Mo_x_ (x = 0.6, 1.4) Bio-HEAs through osteoblast culture experiments. The results revealed that osteoblasts on the surface of the Ti_1.4_–Zr_1.4_–Nb_0.6_–Ta_0.6_–Mo_0.6_ HEA displayed more pseudopodia and longer focal adhesions compared to commercially pure Ti (Figure 4), indicating excellent biocompatibility compared to commercially pure Ti [32]. This enhancement is likely due to a higher Ti and Zr content in the alloy, resulting in the greater presence of TiO_2_ and ZrO_2_ in the oxide layer and promoting a better biocompatibility of the alloy [32].

The elements used in Bio-HEA, such as Ti, Zr, Nb, Ta, and Mo, are considered biologically inert [65]. Consequently, HEAs composed of these elements also do not exhibit surface bioactivity. Traditional Ti alloys can be surface-modified to impart bioactivity to the alloy surface [65].

In recent years, researchers have prepared carbon nanotube-modified ceramic-polymer coatings on Ti alloys for biomedical applications [66]. During immersion tests in artificial saliva and simulated body fluid solutions, coatings containing multi-walled carbon nanotubes demonstrated excellent bioactivity [66]. Furthermore, sbioactive Ti-based biomedical composite materials were developed by combining traditional biomedical alloys with biomedical ceramics (such as titanium dioxide, zirconium dioxide, hydroxyapatite, or tricalcium phosphate) [67].

Berger et al. conducted surface modification on Ti–Nb–Zr–Hf–Ta HEA to render its surface bioactive [68]. Following anodization, the surface of the Ti–Nb–Zr–Hf–Ta alloy exhibited ordered nanotubes. Subsequent pre-calcification treatment on the surface of an anodized Ti–Nb–Zr–Hf–Ta surface resulted in the deposition of Ca–P precipitates. In simulated body fluid immersion tests, substantial hydroxyapatite deposition was observed on the surface of Ti–Nb–Zr–Hf–Ta, indicating surface bioactivity. Therefore, Ti-containing or Ti-rich HEAs can achieve surface bioactivity through surface modification. Although the literature regarding the surface modification of Bio-HEAs remains scarce, this is poised to be a key research direction for the application of HEAs in biomedical implants.

## 5. Biomedical MEAs (Bio-MEAs)

**Figure 5:**
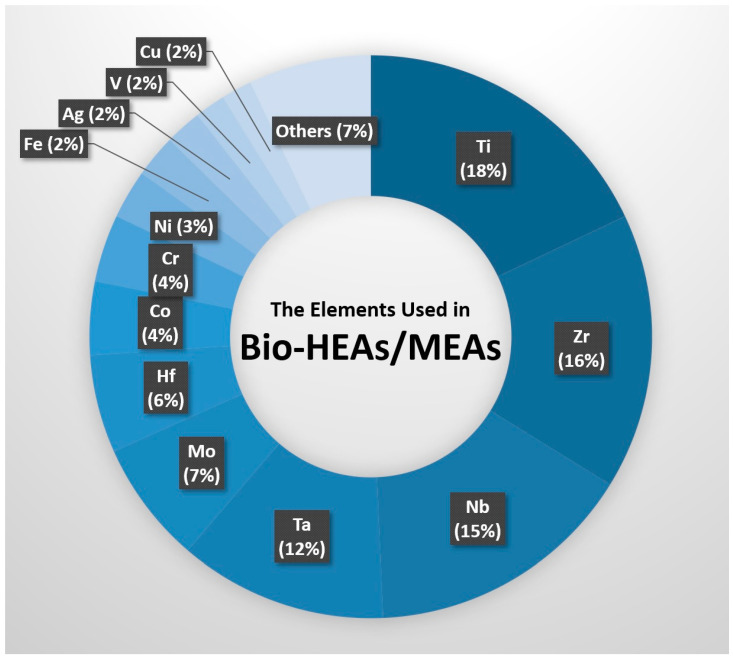
Types and proportions of elements used in biomedical high-entropy alloys/medium-entropy alloys (Bio-HEAs/MEAs) in international journals from 2017 to 2022 (Source: www.webofscience.com).

Most researchers in the field of biomedical HEAs currently prioritize reducing the alloy’s melting point in alloy design, which can be achieved by adjusting the composition of an alloy and adding more low-melting-point elements such as Ti, Zr, Cr, and Co. Adding a significant amount (>30 at%) of Ti in the alloy can effectively prevent segregation and enhance the alloy’s strength, ductility, and biocompatibility, while also reducing the modulus. However, when introducing new alloying elements, one must consider their interactions with other elements, such as mixing enthalpies and differences in atomic radii, which may lead to the formation of intermetallic compounds or precipitates [69]. Therefore, despite the excellent mechanical properties, corrosion resistance, and biocompatibility of HEAs, the challenges of severe compositional segregation and high elastic modulus need to be addressed. Figure 5 shows the elemental ratios used in Bio-HEAs/MEAs published from 2017 to 2022, illustrating that current alloy designs in Bio-HEAs/MEAs are primarily Ti-rich.

In 2018, Nguyen et al. [70] excluded the Mo element in the alloy design, developing a quaternary equiatomic Ti–Zr–Nb–Ta MEA from the five-element Ti–Zr–Nb–Ta–Mo HEA. This adjustment reduced δ from 5.5 to 4.8, decreasing the deformation ability caused by lattice distortion effects. The aim was to achieve an excellent combination of compressive yield strength and deformability for Ti–Zr–Nb–Ta. The results demonstrated a significant increase in compressive strain for Ti–Zr–Nb–Ta (48 ± 6%) compared to Ti–Zr–Nb–Ta–Mo (<9%). Furthermore, the yield strength of the alloy (>1000 MPa) surpassed that of Ti–6Al–4V (970 MPa).

Nguyen et al. [71] subsequently designed a novel non-equiatomic Ti_(25+x)_–Zr_25_–Nb_25_–Ta_(25−x)_ (x = 0, 5, 10, 15, and 20 in at%) MEA, wherein a higher proportion of Ti was incorporated to substitute for Ta in the alloy composition. The influence of δ on the tensile properties of these non-equiatomic Ti–Zr–Nb–Ta alloys was investigated. As-cast Ti_40_–Zr_25_–Nb_25_–Ta_10_ and Ti_45_–Zr_25_–Nb_25_–Ta_5_ exhibited tensile strain greater than 18%, and their tensile strength exceeded 900 MPa. The study revealed that an increase in the value of δ resulted in a higher lattice distortion, leading to an increase in tensile strength but a reduction in ductility for the alloy. Furthermore, in this study, the Pandat simulation system was used to evaluate the solidification gap (liquidus–solidus) of each alloy. It was found that adding more Ti to the alloy could reduce the solidification gap, thereby minimizing the duration during solidification when segregation occurs. Unfortunately, the Ti_45_–Zr_25_–Nb_25_–Ta_5_ with the highest Ti content still exhibited severe compositional segregation.

Mustafi et al. [72], members of Nguyen’s research team, then developed a Ti_35_–Zr_15_–Nb_25_–Ta_25_ MEA with a fixed δ value of 4, aiming to achieve good mechanical properties and biocompatibility. However, the yield strength (842 MPa) and ductility (17%) of Ti_35_–Zr_15_–Nb_25_–Ta_25_ were found to be lower than the previously developed Ti_45_–Zr_25_–Nb_25_–Ta_5_ alloy [71].

**Figure 6:**
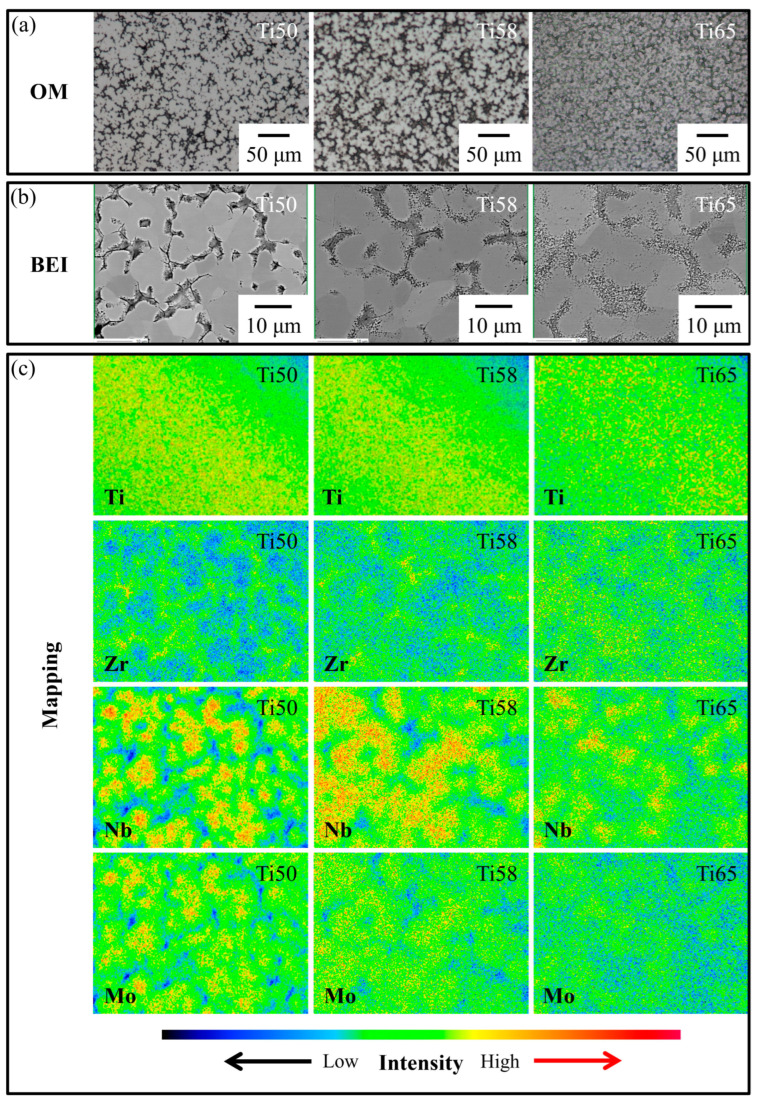
Microstructures of as-cast Ti-rich MEAs: Ti_50_–Zr_25_–Nb_15_–Mo_10_ (Ti50), Ti_58_–Zr_23_–Nb_12_–Mo_7_ (Ti58), and Ti_65_–Zr_20_–Nb_10_–Mo_5_ (Ti65). (**a**) Optical micrographs, (**b**) backscattering electron images (BEI), and (**c**) element mapping images obtained through electron microprobe analysis using wavelength dispersive spectrometers [73]. (Reprinted with permission from Elsevier: J. Alloys Compd. Copyright 2023, License: 5655690226569).

Wong et al. [73] developed three non-equiatomic Ti–Zr–Nb–Mo biomedical MEAs, namely Ti_50_–Zr_25_–Nb_15_–Mo_10_, Ti_58_–Zr_23_–Nb_12_–Mo_7_, and Ti_65_–Zr_20_–Nb_10_–Mo_5_. Through thermodynamic theory and experiments, it was found that adding more Ti to the alloy effectively reduced the elastic moduli of the alloys. The Ti_65_–Zr_20_–Nb_10_–Mo_5_ alloy with the highest Ti content exhibited the lowest elastic modulus (86 GPa). Moreover, the yield strength (1381 MPa) of the alloy was significantly higher than that of conventional biomedical alloys (Ti–6Al–4V ELI, 316L, and Co–Cr–Mo) due to the lattice distortion effect. Furthermore, increasing the Ti content in the alloy effectively reduced the alloy’s melting point, alleviating the segregation present in the HEAs/MEAs (Figure 6) [73]. 

Recently, Son et al. [74] utilized Thermo-Calc simulation software and thermodynamic calculation to develop three MEAs with a single-phase BCC structure: Ti–V–Mo, Ti–Zr–Mo, and Ti–V–Zr–Mo. However, the Ti–Zr–Mo exhibited two different lattice constants of the BCC phase due to severe compositional segregation. Additionally, the Ti–V–Zr–Mo presented a three-phase structure (BCC_1_ + BCC_2_ + Laves) due to the combined influence of severe compositional segregation and a high value of δ. Under the adverse effects of severe compositional segregation and the Laves phase, the true compressive plasticities of Ti–Zr–Mo and Ti–V–Zr–Mo were less than 0.07, making them challenging for practical processing and applications.

## 6. Metastable β Ti-Rich HEAs/MEAs

Bio-HEAs/MEAs demonstrate remarkably high yield strength and corrosion resistance due to the four major effects of HEAs. However, their elastic moduli remain high. Conversely, metastable β-Ti alloys exhibit an extremely low elastic modulus but also low yield strength. By combining the advantages of both kinds of alloys, a metastable β HEA/MEA with simultaneously high yield strength and low elastic modulus can be achieved.

In 2017, Lilensten et al. [75] developed a Ti-rich metastable HEA, Ti_35_–Zr_27.5_–Hf_27.5_–Nb_5_–Ta_5_ by using the Bo-Md theory. The Bo and Md values of Ti_35_–Zr_27.5_–Hf_27.5_–Nb_5_–Ta_5_ were 2.99 and 2.73, respectively. Furthermore, X-ray diffraction results showed a transformation from a single β phase to a β + α” phase in the Ti_35_–Zr_27.5_–Hf_27.5_–Nb_5_–Ta_5_ after tensile testing, indicating stress-induced martensitic transformation, indicating the alloy exhibited a metastable β phase. The yield strength, elastic modulus, and elongation of the solid-solution Ti_35_–Zr_27.5_–Hf_27.5_–Nb_5_–Ta_5_ were 540 MPa, 79 GPa, and 23%, respectively. These research findings demonstrate that the methodology traditionally employed in the development of Ti alloys can be effectively applied to Ti-rich HEAs.

Yuan et al. [76] developed a series of Ti–Zr–Hf–Nb–Ta HEAs/MEAs and observed the following phase formations based on VEC: a single BCC phase when VEC ≥ 4.18, the coexistence of BCC and HCP phases for 4.09 ≤ VEC < 4.18, and a single HCP phase when VEC < 4.09. Notably, the Ti_45_–Zr_45_–Nb_5_–Ta_5_ MEA with a VEC value of 4.18 exhibited a low elastic modulus of 57 GPa. However, the relationship between the elastic modulus and phase stability of Ti_45_-Zr_45_–Nb_5_–Ta_5_ was not discussed in the literature.

Li et al. [77] adopted first-principles calculations to develop the Ti_30_–(NbTaZr)_60_–Mo_10_ HEA. In the calculations, the alloy exhibited the maximum bulk modulus/shear modulus ratio, Poisson’s ratio, and (C_12_–C_44_) values, predicting that the alloy could have high toughness and a low elastic modulus. However, the authors did not present the elastic modulus of the alloy in their results, nor did they discuss relevant modulus data.

**Figure 7:**
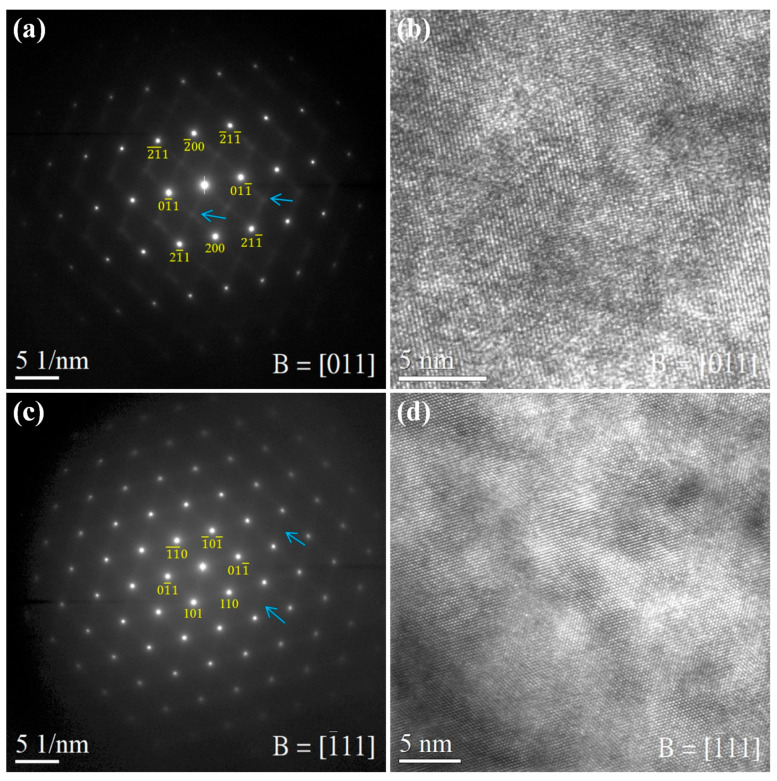
TEM images and SAED patterns of metastable Ti-rich MEA (Ti_65_–Zr_18_–Nb_16_–Mo_1_). (**a**) SAED of the [011] zone axes, (**b**) HR-TEM image in [011] direction (additional streaking is marked by blue arrows), and (**c**) SAED of the [111] zone axes, and (**d**) HR-TEM image in [111] direction (additional streaking is marked by blue arrows) [78]. (Reprinted with permission from Springer Nature: Met. Mater. Int. Copyright 2023, License: 501856029).

**Figure 8:**
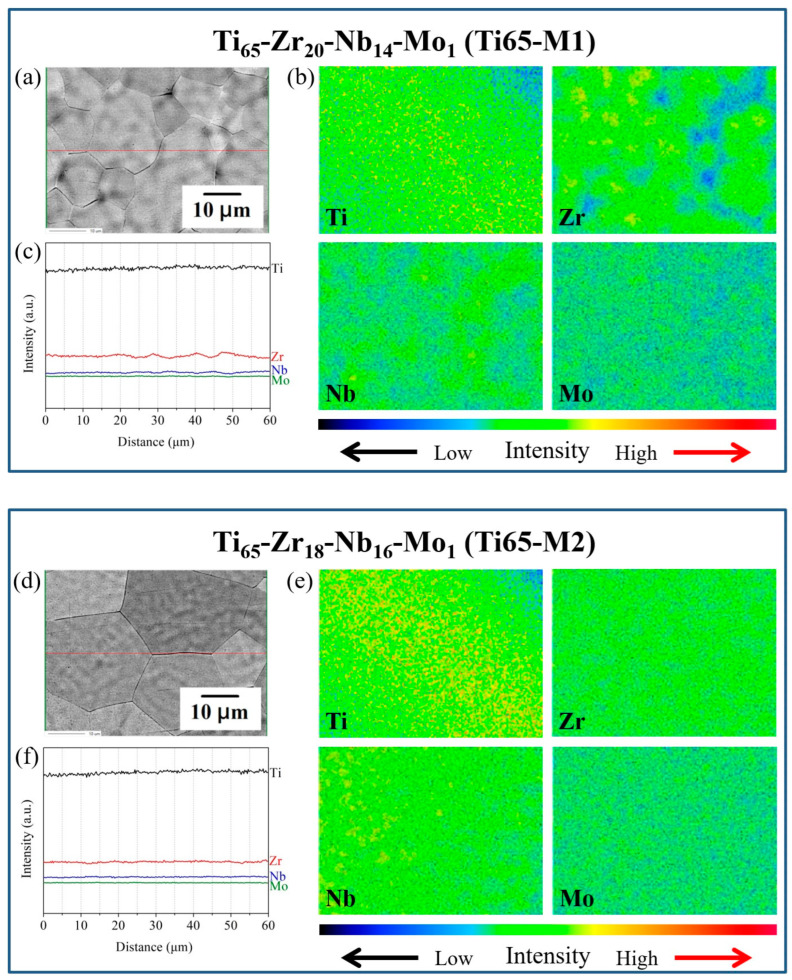
Microstructures and elemental analyses of as-cast Ti_65_–Zr_20_–Nb_14_–Mo_1_ and Ti_65_–Zr_18_–Nb_16_–Mo_1_. (**a**,**d**) backscattering electron images, (**b**,**e**) element mapping images obtained through electron microprobe analysis using wavelength dispersive spectrometers, and (**c**,**f**) line scan curves obtained through an electron microprobe analysis using wavelength dispersive spectrometers [78]. (Reprinted with permission from Springer Nature: Met. Mater. Int. Copyright 2023, License: 501856029).

In recent years, some studies have indicated that the [Mo]_eq_ theory can predict the phase stability of BCC/HCP solid solutions in HEAs [44,61]. Wong et al. [78] focused on Ti_65_–Zr_20_–Nb_10_–Mo_5_, a Ti-rich MEA previously developed. By utilizing the [Mo]_eq_ theory to adjust the alloy composition, they successfully developed two metastable MEAs (Ti_65_–Zr_20_–Nb_14_–Mo_1_ and Ti_65_–Zr_18_–Nb_16_–Mo_1_) ([Mo]_eq_ ≈ 10) with both high yield strength (>1100 MPa) and a lower elastic modulus (~60 GPa). The study confirmed that the phase structure of Ti_65_–Zr_18_–Nb_16_–Mo_1_ was in a metastable state based on the result of SAED-TEM (Figure 7). Furthermore, they proposed that reducing the alloy’s δ value significantly minimized compositional segregation, a highly favorable aspect for subsequent processing (Figure 8).

### Relationships between Thermodynamic Parameters and Modulus

**Figure 9:**
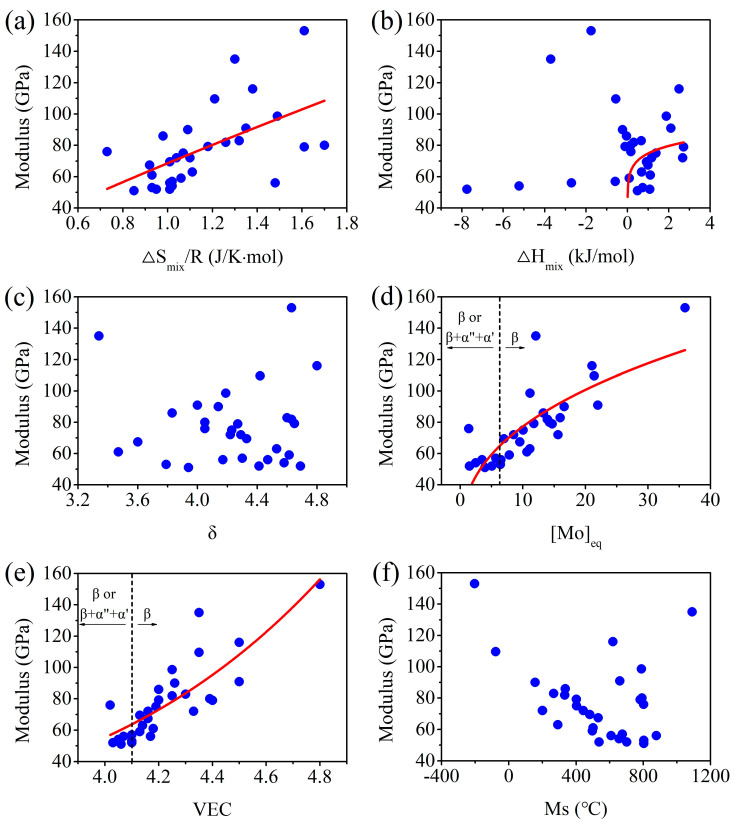
Relationships between the moduli and thermodynamic parameters, as well as between the moduli and phase stability parameters, of biomedical high-entropy alloys/medium-entropy alloys [51,70,72,73,75,76,78,79,80,81,82,83,84,85,86,87]. The red line indicates data trend. (**a**) modulus vs. ΔS_mix_/R, (**b**) modulus vs. ΔH_mix_, (**c**) modulus vs. δ, (**d**) modulus vs. [Mo]_eq_, (**e**) modulus vs. VEC, and (**f**) modulus vs. M_s_.

Figure 9a–c depict the relationships between the moduli and various thermodynamic parameters of published Bio-HEAs/MEAs [51,70,72,73,75,76,78,79,80,81,82,83,84,85,86,87]. From Figure 9a, it can be seen that reducing the ΔS_mix_ values of Bio-HEAs/MEAs effectively lowers the modulus, attributed to the design with non-equiatomic compositions resulting in a lower modulus (see “Section 4.2”). In addition, the moduli of Bio-HEAs/MEAs show a trend with changing ΔH_mix_ values. In Figure 9b, it is evident that the ΔH_mix_ values for most (<80 GPa) Bio-HEAs/MEAs with a low elastic modulus are close to 0, attributed to this design preventing the formation of precipitates and intermetallic compounds. Furthermore, due to the lower ΔS_mix_ values of non-equiatomic MEAs, ΔH_mix_ values approaching 0 or lower contribute to decreasing ΔG_mix_ values (ΔG_mix_ = ΔH_mix_ − TΔS_mix_). On the other hand, the moduli of published Bio-HEAs/MEAs with various values of δ seem not show a clear trend.

Figure 9d–f present the relationship between the moduli and various phase stability parameters of published Bio-HEAs/MEAs [51,70,72,73,75,76,78,79,80,81,82,83,84,85,86,87]. As [Mo]_eq_ and VEC decrease, a noticeable decrease can be observed in the alloy’s elastic modulus. Interestingly, for some HEAs/MEAs, [Mo]_eq_ and VEC fall below the stability limit of the β phase in the metastable theory, suggesting that the high-entropy effect enhances the stability of the β phase. According to statistical results, when [Mo]_eq_ values are below 10 or VEC values range from 4.0 to 4.2, HEAs/MEAs can have an ultra-low elastic modulus (<60 GPa), suggesting that the phase structure of those HEAs/MEAs has reached a metastable state. On the other hand, there is no clear trend between M_s_ values and the moduli of HEAs/MEAs, indicating that the M_s_ theory may not be applicable to HEAs/MEAs.

**Figure 10:**
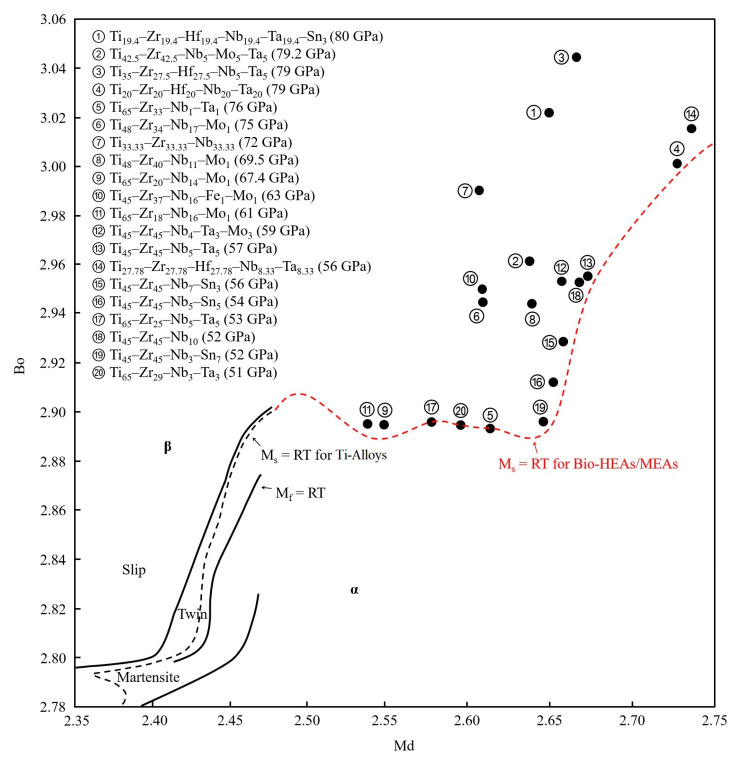
Bo-Md diagram of biomedical high-entropy alloys/medium-entropy alloys (Bio-HEAs/MEAs) with a low elastic modulus (<80 GPa) [75,76,78,80,81,82,83,84,85,86,87].

Figure 10 depicts the position of Bio-HEAs/MEAs with low elastic moduli (<80 GPa) on the Bo-Md diagram [75,76,78,80,81,82,83,84,85,86,87]. The red dashed line in the figure is the M_s_ = RT curve for Bio-HEAs/MEAs, which is based on the Bo and Md values of 20 kinds of Bio-HEAs/MEAs with a low elastic modulus (<80 GPa). In contrast to conventional Ti alloys with a composition constraint (Ti > 50 wt%), the Bo and Md values of HEAs/MEAs are not located near the M_s_ = RT curve. Among them, three alloys (No. 5, 17, and 20) exhibit a β + α′ + α″ phase, while the other Bio-HEAs/MEAs consist of a single β phase. Table 1 presents the thermodynamic parameters, mechanical properties, and phase stability parameters for published Bio-HEAs/MEAs [32,44,51,64,70,72,73,74,75,76,77,78,79,80,81,82,83,84,85,86,87].

## 7. Conclusions

Over the past four decades, the metastable β-Ti alloys have attracted interest in the biomaterials field due to their significantly low modulus. However, even for the metastable β-Ti alloys, an inherent correlation between a low modulus and low yield strength persists. The challenge is in achieving both a low elastic modulus and high yield strength, which are essential for implant materials. Enhancing the yield strength of the alloy contributes to improved fatigue resistance, thereby preventing early implant failure in clinical use. In recent years, several Bio-HEAs/MEAs composed of biocompatible elements such as Ti, Zr, Nb, Ta, and Mo have been developed. However, the alloy design of MEAs poses unique challenges when compared to metastable β-Ti alloys. Despite the contribution of the HEA’s four core effects, the Bio-HEA/MEA exhibits excellent mechanical strength, corrosion resistance, and biocompatibility, but its elastic modulus is also exceptionally high. To meet the needs of biomedical implants, researchers attempt to combine the advantages of HEAs and metastable β-Ti alloys, ultimately developing metastable HEAs/MEAs with high strength and a low modulus. This review focuses on the design from β-Ti alloys to novel metastable HEAs/MEAs, exploring their potential advantages and challenges in biomedical applications. Through a comprehensive comparative analysis of different materials, the potential of HEAs/MEAs and their applications in the biomedical field is envisioned.

## Figures and Tables

**Table 1:** Thermodynamic parameters, mechanical properties, and phase stability parameters of published biomedical high-entropy alloys/medium-entropy alloys [32,44,51,64,70,72,73,74,75,76,77,78,79,80,81,82,83,84,85,86,87].

Alloy	Phase	δ	ΔS_mix_/R (J/K·mol)	ΔH_mix_ (kJ/mol)	Test Methods	σ_y_ (MPa)	E (GPa)	[Mo]_eq_	VEC	Bo	Md	M_s_	Ref.
Ti_33.33_–V_33.33_–Mo_33.33_	β	3.97	1.1	0	Compression	1116	N/A	68.62	4.12	2.886	2.093	–1258	[74]
Ti_30_–Nb_20_–Ta_20_–Zr_20_–Mo_10_	β1 + β2	4.11	1.55	0	Compression	1132	N/A	27.82	4.6	3.009	2.508	214	[77]
Ti_33.33_–Zr_16.67_–Nb_16.67_–Ta_16.67_–Mo_16.67_	β1 + β2	4.23	1.56	–1.33	Compression	1600	N/A	32.84	4.67	2.996	2.458	–87	[64]
Ti_45_–Zr_25_–Nb_25_–Ta_5_	β	4.58	1.2	2.14	Tensile	790	N/A	15.13	4.3	2.959	2.567	432	[70]
Ti_30.5_–Zr_30.5_–Nb_13_–Ta_13_–Mo_13_	β	4.87	1.52	–0.74	Compression	1250	N/A	26.27	4.52	3.002	2.540	36	[32]
Ti_28.33_–Zr_28.33_–Hf_28.33_–Nb_6.74_–Ta_6.74_–Mo_1.55_	β	5.17	1.5	0.99	Tensile	780	N/A	6.68	4.17	3.014	2.732	828	[44]
Ti_33.33_–Zr_33.33_–Mo_33.33_	β1 + β2	5.94	1.1	–1.78	Compression	1598	N/A	40.82	4.67	2.979	2.447	–1116	[74]
Ti_25_–V_25_–Zr_25_–Mo_25_	β1 + β2 + Laves	6.77	1.39	–2.5	Compression	1623	N/A	46.73	4.03	2.936	2.304	–711	[74]
Ti_20_–Zr_20_–Nb_20_–Ta_20_–Mo_20_	β1 + β2	4.63	1.61	–1.76	Compression	1390	153	35.93	4.8	3.036	2.459	–204	[51]
Ti_15_–Zr_15_–Nb_35_–Ta_35_	β	3.34	1.3	–3.71	Tensile	970	135	12.09	4.35	3.066	2.541	1091	[76]
Ti_25_–Zr_25_–Nb_25_–Ta_25_	β	4.8	1.38	2.5	Compression	1100	116	21.04	4.5	3.030	2.584	619	[70]
Ti_50_–Zr_25_–Nb_15_–Mo_10_	β	4.42	1.21	–0.56	Bending	1754	101	21.39	4.35	2.938	2.517	–77	[73]
Ti_37.5_–Zr_25_–Ta_12.5_–Hf_12.5_–Nb_12.5_	β	4.19	1.49	1.88	Nano-indentation	N/A	99	11.16	4.25	2.987	2.642	789	[79]
Ti_35_–Zr_15_–Nb_25_–Ta_25_	β	4	1.35	2.1	Tensile	842	91	21.97	4.5	3.000	2.535	660	[72]
Ti_58_–Zr_23_–Nb_12_–Mo_7_	β	4.14	1.09	–0.24	Bending	1548	90	16.61	4.26	2.914	2.522	157	[73]
Ti_65_–Zr_20_–Nb_10_–Mo_5_	β	3.83	0.98	–0.04	Bending	1381	86	13.27	4.2	2.894	2.518	335	[73]
Ti_37.5_–Zr_37.5_–Nb_15_–Mo_5_–Ta_5_	β	4.6	1.32	0.67	Tensile	925	83	15.97	4.3	2.979	2.606	266	[80]
Ti_40_–Zr_40_–Nb_10_–Mo_5_–Ta_5_	β	4.63	1.26	0.31	Tensile	917	82	13.91	4.25	2.971	2.619	333	[80]
Ti_42.5_–Zr_42.5_–Nb_5_–Mo_5_–Ta_5_	β	4.65	1.18	–0.11	Tensile	911	79.2	11.78	4.2	2.963	2.633	402	[80]
Ti_35_–Zr_27.5_–Hf_27.5_–Nb_5_–Ta_5_	β	4.13	1.38	0.6	Tensile	540	79	4.153	4.1	2.990	2.730	913	[75]
Ti_20_–Zr_20_–Hf_20_–Nb_20_–Ta_20_	β	4.27	1.61	2.72	Compression	926	79	14.69	4.4	3.046	2.662	781	[81]
Ti_65_–Zr_33_–Nb_1_–Ta_1_	β + α″ + α′	4.05	0.73	0.17	Bending	1150	76	1.36	4.02	2.894	2.608	802	[82]
Ti_48_–Zr_34_–Nb_17_–Mo_1_	β	4.23	1.07	1.38	Compression	666	75	10.06	4.19	2.946	2.604	403	[83]
Ti_33.33_–Zr_33.33_–Nb_33.33_	β	4.22	1.1	2.67	Tensile	830	72	15.62	4.33	2.991	2.601	200	[84]
Ti_48_–Zr_40_–Nb_11_–Mo_1_	β	4.33	1.01	0.93	Compression	629	69.5	7	4.13	2.945	2.634	482	[83]
Ti_65_–Zr_20_–Nb_14_–Mo_1_	β	3.6	0.92	0.99	Bending	1188	67.4	9.53	4.16	2.896	2.542	531	[78]
Ti_45_–Zr_37_–Nb_16_–Fe_1_–Mo_1_	β	4.53	1.11	0.69	Tensile	703	63	11.14	4.14	2.950	2.604	291	[85]
Ti_65_–Zr_18_–Nb_16_–Mo_1_	β	3.47	0.93	1.11	Bending	1118	61	10.66	4.18	2.896	2.532	502	[78]
Ti_45_–Zr_45_–Nb_4_–Ta_3_–Mo_3_	β	4.61	1.06	0.09	Tensile	821	59	7.84	4.13	2.954	2.653	497	[86]
Ti_45_–Zr_45_–Nb_5_–Ta_5_	β	4.3	1.02	–0.59	Tensile	690	57	5.7	4.1	2.956	2.669	676	[76]
Ti_27.78_–Zr_27.78_–Hf_27.78_–Nb_8.33_–Ta_8.33_	β	4.17	1.48	0.93	Compression	834	56	6.52	4.17	3.016	2.734	878	[81]
Ti_45_–Zr_45_–Nb_7_–Sn_3_	β	4.47	1.01	–2.71	Tensile	720	56	3.49	4.07	2.930	2.654	608	[87]
Ti_45_–Zr_45_–Nb_5_–Sn_5_	β	4.58	1.02	–5.23	Tensile	750	54	2.48	4.05	2.913	2.648	655	[87]
Ti_65_–Zr_25_–Nb_5_–Ta_5_	β + α″ + α′	3.79	0.93	0.74	Bending	530	53	6.43	4.1	2.897	2.572	803	[82]
Ti_45_–Zr_45_–Nb_10_	β	4.41	0.95	1.08	Tensile	678	52	5.04	4.1	2.954	2.664	537	[86]
Ti_45_–Zr_45_–Nb_3_–Sn_7_	β	4.69	1.01	–7.75	Tensile	790	52	1.48	4.03	2.897	2.641	702	[87]
Ti_65_–Zr_29_–Nb_3_–Ta_3_	β + α″ + α′	3.94	0.85	0.48	Bending	760	51	3.96	4.06	2.896	2.590	802	[82]

## Data Availability

Not applicable.
